# HARKing, transparency, and honesty in reporting: improving reporting of scientific articles

**DOI:** 10.2340/17453674.2026.46651

**Published:** 2026-09-03

**Authors:** Aleksi Reito, Robin Christensen, Soren Overgaard

**Affiliations:** 1 Center for Musculoskeletal Diseases, Tampere University Hospital, Tampere and Coxa Hospital for Joint Replacement, Tampere, Finland; 2 Section for Biostatistics and Evidence-Based Research, the Parker Institute, Bispebjerg and Frederiksberg Hospital, Copenhagen, and Research Unit of Rheumatology, Department of Clinical Research, University of Southern Denmark, Odense University Hospital, Odense, Denmark; 3 Copenhagen University Hospital Bispebjerg Frederiksberg, Department of Orthopaedic Surgery and Traumatology, Copenhagen, and Department of Clinical Medicine, University of Copenhagen, Copenhagen, Denmark

In clinical research, the credibility of results should not depend on how sophisticated the statistics are but on the basis of having a relevant research question, the selection of appropriate outcomes, and the honesty of the scientific process and reporting. Orthopedics makes no exception, as large registries and routinely collected healthcare data have become common in our field. With these analytical opportunities and flexibilities in observational data come a recurring methodological problem known as HARKing, short for hypothesizing after the results are known [1]. HARKing compromises transparency, inflates apparent evidential support for findings, and contributes to potentially misleading conclusions.

The issue concerns the distinction between hypothesis-confirming and hypothesis-generating research, though this distinction is better understood as a continuum rather than a binary divide [2]. The evidentiary strength of an observational study depends on the degree to which its hypothesis, outcomes, exposures, and analysis plan were specified before the data was examined. A study with a pre-registered protocol, a prospectively defined statistical analysis plan, and data collected specifically to address a single question sit at the confirmatory end of this continuum and can provide strong, credible evidence. In that example, a hypothesis could be the driver of a research question. At the other end sits the unregistered, retrospective analysis of convenience data, where multiple hypotheses could have been tested and the reported one selected after inspection of results. Here the evidence is inherently weak, regardless of the P value. Most observational studies fall somewhere between these poles, and their findings should be interpreted accordingly [3].

HARKing occurs when researchers analyze data first and then propose or construct a hypothesis that fits the observed results, while presenting that hypothesis as if it had been specified beforehand [4]. Authors may even outline the main finding in the title. From the outside, the study appears theory-driven and focused. However, the hypothesis is a post-hoc explanation shaped by the data itself. This practice is tempting because statistically significant findings naturally invite interpretation, and surgeons are good at matching the finding with a priori beliefs. However, the statistical framework used to assess significance assumes that the hypothesis existed before the test. When this assumption is violated, P values and confidence intervals no longer mean what readers think they mean [5].

**Infobox**

- Pre-register possible hypotheses and analysis plans
- Clearly separate confirmatory and exploratory analyses
- Use blinded data analysis when possible
- Document all tested hypotheses and models; distinguish main from sensitivity analyses
- Involve an independent statistician or methodologist

The main problem with HARKing is not only deliberate deception but also distorted inference. When many analyses are performed and only the most interesting results are highlighted, the risk of false positives increases substantially [6]. An association that appears strong may simply reflect chance, especially when many variables and outcomes are analyzed [7]. Presenting such findings as confirmatory gives them a level of credibility they have not earned. This contributes to poor reproducibility, as results derived from extensive data exploration often fail to replicate in independent datasets [8].

The risk is particularly high in research areas with extensive registry data, such as orthopedics and traumatology. This allows researchers to test an enormous number of questions with relative ease. National registers, arthroplasty databases, and administrative health records contain thousands of variables and long follow-up periods. Outcomes such as revision surgery or complications can be defined in multiple ways. Exposures can be categorized, transformed, or combined. Each analytical choice adds flexibility, and with flexibility comes the risk of overinterpreting chance findings. When a statistically significant result is found, it can be tempting to frame it retrospectively as the answer to a pre-existing clinical question [7].

Closely related to HARKing are the concepts of SHARKing and THARKing [9]. SHARKing, or secretly hypothesizing after results are known, refers to situations where researchers are aware that a hypothesis was generated after seeing the data but do not disclose this in the manuscript. The study is written in a confirmatory tone, giving readers the impression of strong a priori reasoning, which is misconduct. THARKing, or transparently hypothesizing after results are known, takes a different approach. Here, authors openly state that hypotheses emerged during data exploration and that the findings are exploratory. THARKing preserves scientific integrity and allows readers to interpret results appropriately. Importantly, THARKing is not a flaw; it is an honest description of how many valuable research ideas are born.

In orthopedics, registry studies can influence clinical guidelines, implant selection, and healthcare policy. Overconfident conclusions based on exploratory analyses risk promoting ineffective or even harmful practices. They can also divert attention and resources away from truly confirmatory studies that are needed to establish causality. When exploratory findings are later contradicted, trust in research may erode, even if the original issue was one of reporting rather than data quality.

Transparency is therefore the central ethical principle in avoiding HARKing. This does not mean that all studies must be preregistered or that exploratory analyses should be discouraged. Rather, it requires clear communication about what was planned and what was discovered along the way. Readers should be able to distinguish between hypotheses that were tested and patterns that were observed. Language matters: terms such as “exploratory,” “post hoc,” and “hypothesis-generating” provide important context and should not be seen as weakening a study. On the contrary, they signal methodological honesty.

In a field increasingly driven by large datasets, orthopedic research must resist the temptation to frame exploratory analyses as confirmatory evidence. Registries and routinely collected observational data are invaluable for hypothesis generation, signal detection, and prioritization of future research, but they rarely provide definitive (causal) answers in isolation. Greater emphasis on prespecified protocols, transparent analytical decisions, reproducible methods, and external validation would strengthen the credibility and utility of such research [5]. Exploratory findings should therefore be presented for what they are: an essential step toward more rigorous evaluation, ideally through well-designed randomized trials and independent replication [8].

Ultimately, HARKing is a reminder that good science is not just about results, but about the path taken to reach them. Clear separation between hypothesis generation and hypothesis testing does not slow progress; it strengthens it. By openly acknowledging uncertainty and being honest about how hypotheses arise, orthopedic research can make better use of its rich data resources and produce knowledge that is both credible and clinically meaningful.

## Notes

Conception or design of the work: RC and AR. Acquisition, analysis, or interpretation of data for the work and drafting the work or revising it critically for important intellectual content: all authors. The authors confirm that they have approved the final version of the manuscript and agree to be accountable for all aspects of the work, ensuring that any questions related to the accuracy or integrity of any part of the work are appropriately investigated and resolved. All individuals listed as authors meet the criteria for authorship, and no one who qualifies for authorship has been omitted.

AI statement – During the preparation of this work the authors used ChatGPT from OpenAI to improve spelling and wording. After using this tool/service for individual pieces, the authors reviewed and edited the content as needed and take full responsibility for the content of the publication.

Conflict of interests – Complete disclosure of interest forms according to ICMJE are available on the article page, doi: 10.2340/17453674.2026.46651
